# Expression of toll-like receptor 4 and its associated cytokines from peripheral blood mononuclear cells in Leghorn chickens

**DOI:** 10.14202/vetworld.2023.1541-1545

**Published:** 2023-07-31

**Authors:** Juthatip Jeenkeawpieam, Chananphat Tantikositruj, Warangkana Kitpipit, Anyarat Thiptara, Autchara Kayan, Kittichai Unjit, Siriluk Sintupachee, Chaiwat Boonkaewwan

**Affiliations:** 1 Akkhraratchakumari Veterinary College, Walailak University, Nakhon Si Thammarat 80160, Thailand; 2 Department of Livestock Development, National Institute of Animal Health, Bangkok 10900, Thailand; 3 Department of Livestock Development, Veterinary Research and Development Center (Upper Southern Region), Nakhon Si Thammarat 80110, Thailand; 4 Department of Animal Science, Kasetsart University, Bangkok 10900, Thailand; 5 Program in Creative Innovation in Science and Technology, Faculty of Science and Technology, Nakhon Si Thammarat Rajabhat University, Nakhon Si Thammarat, 80280, Thailand; 6 One Health Research Center, Walailak University, Nakhon Si Thammarat, 80160, Thailand

**Keywords:** Leghorn chicken, peripheral blood mononuclear cell, pro-inflammatory cytokine, Toll-like receptor 4

## Abstract

**Background and Aim:**

Immune cells require toll-like receptor 4 (TLR4) to respond to lipopolysaccharides (LPS) by releasing pro-inflammatory cytokines. Peripheral blood mononuclear cells (PBMCs) are used to assess changes in cytokines released in response to diseases or pathogens. This study aimed to assess TLR4 gene expression in PBMCs from Leghorn chicken and the release of related cytokines.

**Materials and Methods:**

Peripheral blood mononuclear cells were isolated from blood samples obtained from Leghorn chicks. The PBMC cultures were stimulated with various concentrations of LPS (0.01-1 μg/ml). Polymerase chain reaction was used to detect TLR4 expression. The production of tumor necrosis factor-alpha (TNF-α) and interleukins (IL-1β and IL-6) was quantified using an enzyme-linked immunosorbent assay.

**Results:**

We found that TLR4 was expressed in both non-stimulated and stimulated Leghorn chicken PBMCs. In addition, the release of TNF-α, IL-1β, and IL-6 levels in Leghorn chicken PBMCs increased significantly with an increase in LPS concentration (0.01–1 μg/mL) (p < 0.05).

**Conclusion:**

Although TLR4 was expressed in both non-stimulated and stimulated Leghorn chicken PBMCs, its expression was significantly higher in LPS-stimulated PBMCs Therefore, the chicken’s endotoxin response can be assessed by evaluating the pro-inflammatory cytokine production from PBMCs.

## Introduction

Toll-like receptors (TLRs) are expressed in multiple cellular compartments in various organs and cells [1, 2]. It is crucial for the innate immune response, which recognizes pathogens based on their characteristic molecules, including lipopolysaccharides (LPS) [3]. Like mammals, pathogens induce an inflammatory response in birds by interacting with their cellular and soluble pattern recognition receptors [4, 5]. Toll-like receptor 4 is necessary to release pro-inflammatory cytokines from immune cells, such as monocytes and macrophages, in response to LPS [6, 7].

In chicken farms, feces, feathers, germs, and fungi are serious contaminants. Poultry farm dust contains high levels of endotoxins, including LPS [8]. Peripheral blood mononuclear cells (PBMCs), a diverse population of monocytic and lymphocytic white blood cells, are crucial for innate and adaptive immune responses as they can recognize and control infections [9]. Thus, PBMCs are ideal markers for evaluating changes in the cytokine release associated with disease or pathogen response.

Different chicken breeds exhibit varying degrees of resistance or vulnerability to diseases and pathogens, which causes variations in their immune response during infection [10]. Hence, TLR expression also varies between chicken breeds [11]. Leghorn chickens are frequently employed as layer hens in several countries. They cost-effectively convert feed to white eggs and are usually kept for approximately 2 years at a farm [12].

This study aimed to examine the TLR4 expression in PBMCs to better understand and improve chicken health. In addition, we determined the release of pro-inflammatory cytokines from PBMCs activated with LPS.

## Materials and Methods

### Ethical approval

The study was approved by the Animal Ethics Committee of Kasetsart University (ACKU61-AGR-009).

### Study period and location

The study was conducted from December 2018 to April 2020. For this investigation, hens were provided by the Vajokkasikij Chicken Farm. The samples were processed by the Department of Animal Science in the Faculty of Agriculture at Kasetsart University.

### Animals and blood collection

We collected 3 mL of blood from six Leghorn hens aged 14–18 weeks through wing vein puncture and stored them in tubes containing ethylenediaminetetraacetic acid. These blood samples were used for hematological analysis and PBMC isolation and stimulation.

### Hematological study and PBMC isolation

Hematological abnormalities were analyzed using 0.5 mL of blood. Complete blood count was determined using the automated Vet system (Sysmex 1000V, Norderstedt, Germany). Hematocrit (HCT) was determined manually using micro HCT capillary tubes.

The PBMCs were separated from blood samples by gently stacking 2.5 mL of blood over 3 mL of histopaque solution and centrifuging for 30 min at 400× *g*. The white band containing mononuclear cells was isolated and washed thrice with RPMI 1640 medium (Gibco, Gibthai, Thailand) by centrifuging at 400× g for 5 min. The PBMCs were suspended in RPMI 1640 medium (with 10% heat-inactivated fetal calf serum) and adjusted to a concentration of 2 × 10^6^ cells/mL. The trypan blue dye exclusion technique was used to determine cell viability by combining the cell suspension with 0.4% trypan blue and counting the viable cells using a hemocytometer.

### Peripheral blood mononuclear cells stimulation and measurement of pro-inflammatory cytokines

Peripheral blood mononuclear cells (2 × 10^6^ cells/mL) were cultured in 96-well plates (in duplicates) and separated into the following four groups: (1) Control PBMC and PBMCs stimulated with LPS at (2) 0.01 μg/mL, (3) 0.1 μg/mL, and (4) 1 μg/mL. All four groups were cultured for 24 h at 41°C in a humidified atmosphere containing 5% CO_2_. Then, we collected the culture medium from each well and stored it at −20°C to determine tumor necrosis factor-alpha (TNF-α), interleukins (IL)-1β, and IL-6 levels. On the day of cytokine quantification, the culture media was thawed and prepared in duplicate. Enzyme-linked immunosorbent assay was performed using a kit according to the manufacturer’s instructions (Cloud-Clone Corp, USA) for quantifying TNF-α, IL-1β, and IL-6.

### DNA extraction and polymerase chain reaction (PCR)

We pooled two samples to obtain the PBMCs. The genomic DNA was extracted from four sets of cultured PBMCs using a DNA extraction kit (BiotechRabbit, Germany) following the manufacturer’s instructions. DNA concentration was determined using spectrophotometry at a wavelength of 260 nm and DNA purity was determined using OD_260_/OD_280_ using the NanoDrop (Biodrop, UK). Polymerase chain reaction was conducted using a standard protocol (Green PCR MasterMix, BiotechRabbit) but with a different annealing temperature for each primer (TLR4 and β-actin) (Table-1) [13]. The β-actin gene was used to assess the quality of genomic DNA samples. The PCR reaction mix (12.5 μL) contained 1 μL genomic DNA, 1.25 μL primers, 5 μL ddH_2_O, and 6.25 μL GreenPCR Master Mix(Qiagen, Hilden, Germany). Initial denaturation was performed at 95°C for 5 min, followed by 35 cycles at 95°C for 1 min. The annealing temperature was at 59.5°C for TLR4 and 60.5°C for β-actin for 25 s with a 10 min final extension step at 72°C. The negative control consisted of each sample without the genomic DNA. The PCR products were stored at 4°C, and 10 μL was used for electrophoresis (1.5% agarose gel). The DNA bands were observed under UV irradiation (Gel Doc XR system, Bio-Rad, California,USA).

**Table-1:** Toll-like receptors primers and annealing temperature.

Target gene	Primer (5’-3’)	Annealing Temp. (°C)	Accession no.
TLR4	F: TGCACAGGACAGAACATCTCTGGA R: AGCTCCTGCAGGGTATTCAAGTGT	59.5	AY064697
Beta-actin	F: GCACCACACTTTCTACAATAG R: ACGACCAGAGGCATACAGG	60.5	L08165

TLR4=Toll-like receptor 4

### Statistical analysis

The data were analyzed using Prism 5 software (GraphPad Software, USA)and are presented as mean ± standard deviation. The statistical significance of the effect of different LPS concentrations on TNF-α, IL-1β, and IL-6 levels in Leghorn PBMCs was tested using a one-way analysis of variance and the student Newman-Keuls test. Statistical significance was defined as p < 0.05.

## Results

### Hematology and cell viability

The hematological values of blood samples collected from six Leghorn chickens were in the normal range (Table-2). The cell viability of the Leghorn chicken PBMCs obtained was 92.93% ± 0.84%.

**Table-2:** Hematology and PBMC viability (n = 6).

Parameter	Leghorn chicken
Red blood cell (10^6^/µL)	2.59 ± 0.26
Hemoglobin (g/dL)	9.89 ± 0.99
Hematocrit (%)	27.92 ± 2.94
White blood cell (cells/mm^3^)	8039.17 ± 1366.90
Heterophil (%)	65.67 ± 5.76
Basophil (%) g	0.67 ± 1.30
Eosinophil (%)	1.08 ± 1.31
Lymphocyte (%)	28.42 ± 3.96
Monocyte (%)	3.67 ± 1.50
PBMC	
Total PBMC (cells)	4.496 ± 0.248 × 10^6^
Cell viability (%)	92.93 ± 0.84

Data are reported as mean ± standard deviation. PBMC=Peripheral blood mononuclear cells

### Expression of TLR4

**Figure-1:**
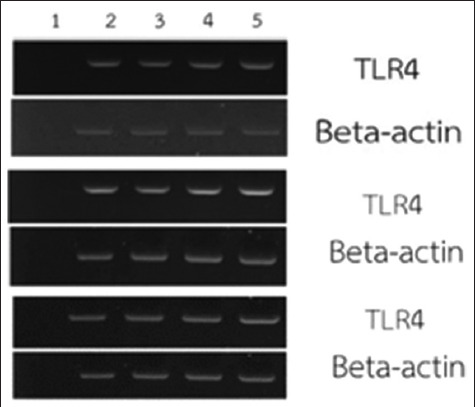
The expression of TLR4 genes in not stimulated and LPS-stimulated PBMC of Leghorn chicken. Lane 1 is negative control, Lane 2 is PBMC only, Lane 3 is LPS (0.01 μg/mL)-stimulated PBMC, Lane 4 is LPS (0.1 μg/mL)-stimulated PBMC, and Lane 5 is LPS (1 μg/mL)-stimulated PBMC. TLR4=Toll-like receptor4, LPS=Lipopolysaccharides, PBMC=Peripheral blood mononuclear cells.

DNA was isolated from the four PBMC sets cultured for 24 h. Toll-like receptor 4 expression was evaluated in the non-stimulated PBMCs and those stimulated with 0.01, 0.1, and 1 μg/mL of LPS (Figure-1).

### Pr-inflammatory cytokine

**Figure-2:**
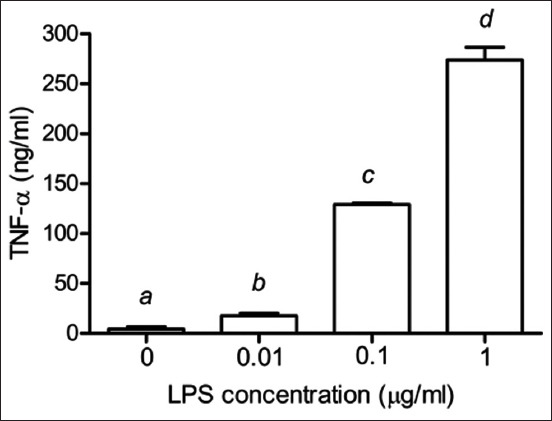
Effects of LPS on the production of TNF-α in PBMCs of Leghorn chickens. Peripheral blood mononuclear cells were cultured with LPS (0.01–1 μg/mL). Data are expressed as the mean ± SD of six independent experiments. ^a,b,c,d^Statistically significant difference in TNF-α release in each column (p < 0.05). LPS=Lipopolysaccharides, TNF-α=Tumor necrosis factor-alpha, PBMC=Peripheral blood mononuclear cells, SD=Standard deviation.

**Figure-3:**
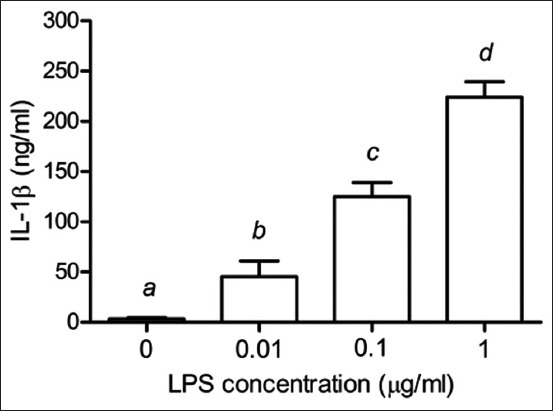
Effects of LPS on the production of IL-1β in PBMCs of Leghorn chickens. Peripheral blood mononuclear cells were cultured with LPS (0.01–1 μg/mL). Data are expressed as the mean ± SD of six independent experiments. ^a,b,c,d^Statistically significant difference in IL-1β release in each column (p < 0.05). LPS=Lipopolysaccharides, IL=Interleukin, PBMC=Peripheral blood mononuclear cells, SD=Standard deviation.

**Figure-4:**
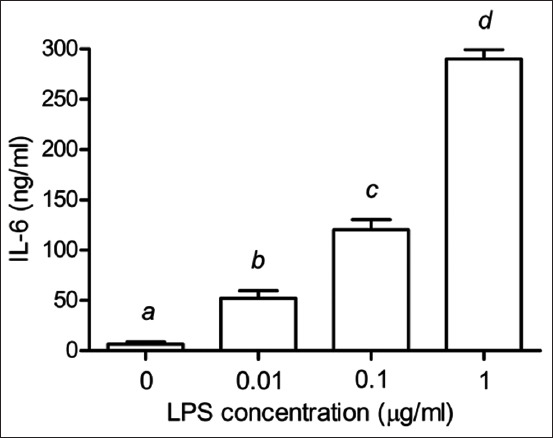
Effects of LPS on the production of IL-6 in PBMCs of Leghorn chickens. Peripheral blood mononuclear cells were cultured with LPS (0.01–1 μg/mL). Data are expressed as the mean ± SD of six independent experiments. ^a,b,c,d^Statistically significant difference in IL-6 release in each column (p < 0.05). LPS=Lipopolysaccharides, IL=Interleukin, PBMC=Peripheral blood mononuclear cells, SD=Standard deviation.

As shown in Figure-2, TNF-α secretion increased significantly in the PBMCs with increasing LPS concentrations (p < 0.05). Similarly, the IL-1β secretion also increased in the PBMCs stimulated with 0–1 μg/mL of LPS in a dose-dependent manner (p < 0.05) (Figure-3). Figure-4 shows the statistically significant increase in IL-6 levels with increased LPS concentrations (p < 0.05).

## Discussion

Hematological values are the primary indicators of the health status of chickens. We found that the hematological characteristics of Leghorn, an egg-laying variety of chicken, were normal, indicating that the chickens were healthy [14]. PBMCs have been extensively employed to study the immune response to pathogens [15]. Cell viability also provides an early indicator of cell quality, as viability rates ≤90% represent good cell quality. We did not observe appreciable differences in the total number of PBMCs between fresh and chilled blood. However, fresh blood PBMCs were more viable than those stored for 24 h, and PBMC viability drastically declined with time [16, 17].

We found that TLR4 was expressed in both non-stimulated and LPS-stimulated PBMCs. In chicken, TLR4 has been identified in leukocytes and other tissues, including the liver, kidneys, lungs, fallopian tubes, small intestine, colon, and spleen. Moreover, TLR4 expression was found in leukocytes and heterophils from various chicken breeds [18, 19]. Toll-like receptor 4 responds to LPS in mammals and birds by activating immune cells, such as monocytes, macrophages, and B cells, that combat infections [20, 21]. A study reported that TLR4 gene expression is elevated at varying levels in different chicken breeds after LPS stimulation [2]. Consistently, our results showed that TLR4 was expressed in both stimulated and non-stimulated PBMCs isolated from Leghorn chickens, suggesting that this might be the case in other chicken breeds as well.

Tumor necrosis factor-alpha participates in various immune functions, including tissue inflammation, pathogen-induced inflammation, apoptosis, and immune response to intestinal bacterial infections, such as salmonellosis [7]. Interleukin-1β, essential for the inflammatory response, is released by LPS-stimulated immune cells, such as phagocytic and splenic cells [22]. The release of pro-inflammatory cytokines (IL-1, IL-6, and TNF) and inflammatory mediators is routinely induced in laboratory experiments using bacterial LPS. Toll-like receptor 4/myeloid differentiation protein-2 complex is produced when LPS forms a covalent bond with TLR4 [23]. In general, infection and disease induce fever, a physiological defensive response mediated by cytokines, such as TNF-α, IL-1β, and IL-6 [24, 25]. Thus, an in-depth understanding of the inflammatory response in Leghorn chicken can help improve health and meat production. In addition, the inhibition of TNF-α, IL-1β, and IL-6 production can be used to assess the anti-inflammatory activities of plant extracts [26].

When PBMCs from various chicken breeds were stimulated with LPS, TLR4 expression increased significantly compared to non-stimulated PBMCs [2]. Studies have revealed that IL-1 and IL-6 are the most abundant cytokines expressed in peripheral blood monocyte-derived dendritic cells exposed to LPS (1 μg/mL) for 24 h [27]. Instead of measuring TLR4 or cytokines expression, we evaluated the downstream products of TLR4 stimulation, including TNF-α, IL-1β, and IL-6. We showed that stimulating PBMCs with various concentrations of LPS increased the secretion of TNF-α, IL-1β, and IL-6 in a dose-dependent manner. Further, 1 μg/mL of LPS was recommended for stimulating the PBMCs isolated from Leghorn chicken to check the TLR4 expression and cytokine production.

Stimulating chicken PBMCs with avian pathogenic *Escherichia coli* (APEC) and APEC mutant *fimH* genes also induced the production of TLRs, such as TLR1/6/10, TLR3, TLR4, TLR5, and TLR7 and release of cytokines, including IL-1β, IL-8, IL-18, and TGF-β [28]. Therefore, this study established that Leghorn chicken PBMCs responded to LPS stimulation by expressing TLR4 and secreting its related cytokines.

## Conclusion

It is concluded that Leghorn chicken PBMCs respond to LPS stimulation by expressing TLR4 and its related cytokines. Thus, the release of pro-inflammatory cytokines by chicken PBMCs might indicate the endotoxin response in chickens.

## Authors’ Contributions

JJ, CT, and WK: Collected the samples and conducted the study. AK and KU: Technical guidance and supervision. AT and SS: Statistical analysis. CB and WK: Designed the study, collected the data, and reviewed the manuscript. All authors have read, reviewed, and approved the final manuscript.
